# Verification of the Efficacy of New Diagnostic Criteria for Retropharyngeal Nodes in a Cohort of Nasopharyngeal Carcinoma Patients

**DOI:** 10.7150/ijms.58375

**Published:** 2021-08-13

**Authors:** Dom-Gene Tu, Hsuan-Yu Chen, Wei-Jen Yao, Yu-Sheng Hung, Yu-Kang Chang, Chih-Han Chang, Yu-Wen Wang

**Affiliations:** 1 Department of Nuclear Medicine, Ditmanson Medical Foundation Chia-Yi Christian Hospital, Chia-Yi, Taiwan.; 2 Institute of Statistical Science, Academia Sinica, Taipei, Taiwan.; 3 Division of Nuclear Medicine, Department of Medical Imaging, Chi Mei Medical Center, Liouying, Tainan, Taiwan.; 4 Department of Medical Imaging Chi Mei Medical Center, Liouying, Tainan, Taiwan.; 5 Department of Biomedical Engineering, National Cheng Kung University, Tainan, Taiwan.; 6 Department of Radiation Oncology, Ditmanson Medical Foundation Chia-Yi Christian Hospital, Chia-Yi, Taiwan.; 7 Department of Radiation Oncology, Chi Mei Medical Center, Liouying, Tainan, Taiwan.

**Keywords:** diagnosis, nasopharyngeal carcinoma, retropharyngeal nodes, radiotherapy

## Abstract

**Purpose:** A multistage approach to diagnose lateral retropharyngeal nodes (LRPNs) of nasopharyngeal carcinoma (NPC) had been proposed and warranted for validation.

**Methods:** Between 2012 and 2017, the patients with newly diagnosed NPC were enrolled. The responsive nodes or those that progressed during follow-up were positive. The criteria for the multistage approach delimited LRPNs with a minimal axial diameter (MIAD) ≥ 6.1 mm were assessed as positive and if the mean standard uptake value ≥ 2.6, or if the maximal coronal diameter ≥ 25 mm and maximal axial diameter ≥ 8 mm with nodes MIAD < 6.1 mm were also considered as positive. The outcomes were compared with the MIAD cutoff value ≥ 6 mm (traditional method). A chi-squared test was used to compare two areas under the curve of the receiver operating characteristic curves.

**Results:** A total of 67 eligible NPC cases and 155 LRPNs (72 positive and 83 negative) were analyzed. The accuracy, specificity, and sensitivity of the traditional method were 0.91, 0.93, and 0.89, respectively. The values for the multistage approach all reached 0.94. The area under the curve was significantly greater for the multistage approach compared with the traditional method (p = 0.023).

**Conclusion:** The results support the advantage of the multistage approach.

## Introduction

The accurate diagnosis of lateral retropharyngeal nodes (LRPNs) with images for nasopharyngeal carcinoma (NPC) is important. Magnetic resonance imaging (MRI) is considered as a golden standard for detecting LRPNs in NPC 1. Based on control subject analysis, Lam and King used minimal axial diameter (MIAD) of 4 and 5 mm as the upper limit of normal LRPNs 2, 3. A diameter of 5 mm or higher as a criterion of malignancy was widely indicated by previous studies 4-12. However, Zhang et al. reported that ≥6 mm diameter might be a better cutoff point for malignancy 13. The method proposed by Zhang was reviewed as a “robust standard methodology” 14. A better clinical prediction was also obtained by Li et al. using a MIAD of 6 mm 15. The newly proposed size criterion for malignant LRPNs minimally up-shifted the definition of LRPN involvement from “≥ 5 mm” to “> 5 mm,” with the consensus of an international guideline for NPC and several publications 16-20. However, based on the prognostic value of LRPN metastasis laterality, a recent study 21 favored ≥5 mm as more suitable than ≥6 mm as the cutoff value of MIAD. The inconsistency of results in the above-published data characterizing LRPNs implies that when only a single factor is used to determine the malignancy of LRPNs, the power of detection could be limited. 2-[ ^18^F]- F-fluorodeoxyglucose (FDG) positron emission tomography (PET)/computed tomography (CT), with its functional imaging sensitivity in detecting cancerous nodal lesions, may be complementary to MRI, which was highly encouraged in the 2017 American Joint Committee on Cancer staging system 22. Previously, we proposed a diagnostic criteria of parameters combined mean standard uptake value (SUV) from FDG PET/CT and MRI (MIAD, maximal axial diameter, and maximal coronal diameter) with a significantly higher reported accuracy. This criterion is still subject to external review despite its higher reported accuracy 23. Using a multistage approach to assess a node in daily practice can be cumbersome. Thus, the superiority of the new criteria must be verified to convince clinicians to adopt them. This study aimed to validate the efficacy of the novel multistage approach to diagnose LRPNs with a new cohort of NPC cases.

## Methods

This study was approved by the Institutional Review Board of the Chi Mei Medical Center (Approval numbers: 10710-L06). Although consent was not specifically obtained for this retrospective review, all information was anonymized and de-identified before its analysis.

### Patients and treatment

Between Oct 2012 and Dec 2017, the patients with newly diagnosed NPC were enrolled. Exclusion criteria included cases without MRI or FDG PET**,** examining dates within 3 weeks and before starting any cancer treatment, cases with incomplete definitive RT dose (under 59.4 Gy), those who did not undergo MRI within 3 months after the end of RT date, those whose initial MRI failed to reveal any LRPN, and those lacking tissue proof of NPC. Patients with additional head and neck cancers or acute inflammation were also excluded from this study. All patients received RT-based cancer treatment (including induction chemotherapy + concurrent chemoradiation, chemoradiation ± adjuvant chemotherapy, or RT alone). RT was given with standard fractionations with dose ranging from 59.4 Gy to 72 Gy, with mean ± standard deviation (SD) = 70.73 ± 1.66 Gy. All the patients received RT with intensity-modulated RT or volumetric arc therapy with an accelerator or TomoTherapy.

### Imaging protocol and assessment

All the patients underwent initial MRI and PET/CT. MRI and FDG PET/CT scans were conducted less than 3 weeks apart (mean ± SD = 2.5 ± 6.0; range: 0-17 days) before cancer treatment. The details of imaging protocols for MRI (Siemens Medical Systems, Erlangen, Germany) and PET/CT (GE Healthcare, Chicago, IL, USA) and the methods of measurement of nodal parameters were identical to those described in our previous reports 23, 24. Three experienced NPC physicians blinded to patients' details evaluated for this study. A radiation oncologist and a nuclear medicine physician evaluated both MRI images and the corresponding FDG PET/CT data and the mean SUV of the FDG PET/CT data for the region of interest using the MRI image as an anatomical reference. Any disagreements were resolved by consensus among the three physicians.

### Follow-up and Assessment of Lymph Nodes

**Figure 1:**
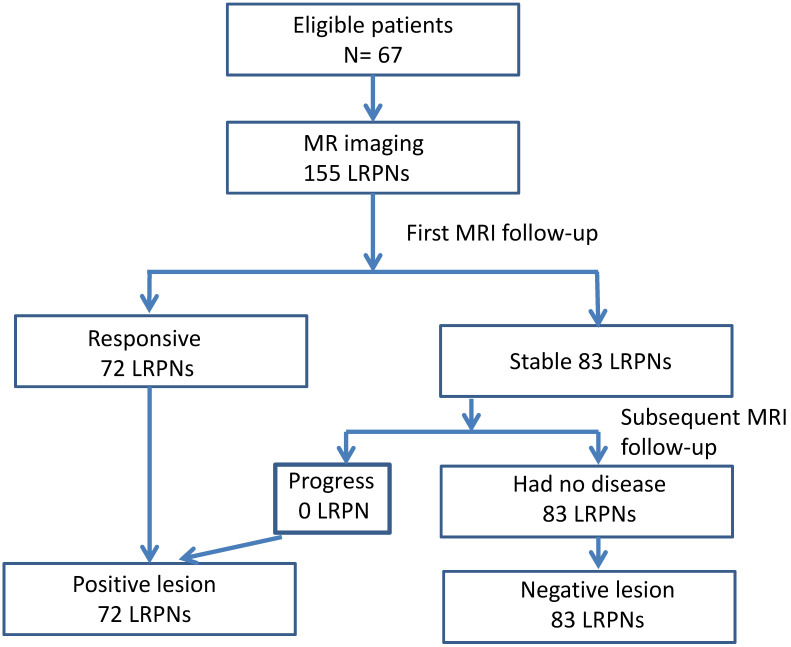
Flowchart outlining the follow-up MRI results of 155 lateral retropharyngeal nodes in 67 patients.

The images of LRPNs were reviewed. We observed the response of these nodes before and after RT-based local treatment by serial MRI. Repeated MRI was performed within 1-2 months (mean ± SD = 43.9 ± 15.7; 6-85 days) after RT. Positive nodes could be identified for patients with a follow-up of fewer than 6 months. The absence of nodal recurrence of more than 6 months was needed if negative nodes were diagnosed. Overall, the follow-up period after RT ranged from 0.2 months to 84.4 months (mean ± SD = 30.1 ± 20.9). We measured the changes in maximal axial diameter and maximal coronal diameter before and after the treatment to determine the nature of nodes 23, 25. The responsive nodes and those that progressed during follow-up were positive; otherwise, the nodes were considered negative (Fig.1). The images of widely accepted characteristics of an involved node, as recommended by international consensus of delineation of target volume for NPC extracapsular extension, central necrosis, and three or more contiguous confluent LRPNs in MRI and overt FDG avid node in PET/CT, were also recorded in addition to the nodal diameter for comparison 16.

**Figure 2:**
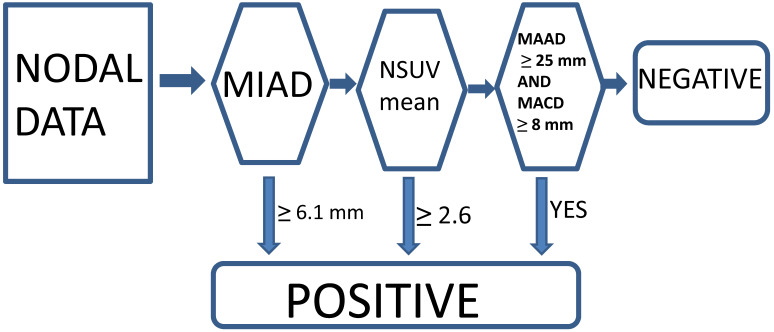
Multistage approach with new criteria for lateral retropharyngeal nodes in retropharyngeal carcinoma.

Currently, we think that the optimal conventional method with a single parameter criterion (MIAD ≥ 6.0 mm) yielded positive results, although we also tested the outcome results by using MIAD ≥ 5.0 mm for comparison 13-15, 23. The proposed new criteria with the multistage approach included MIAD and mean SUV from the FDG-PET and MRI (maximal axial diameter and maximal coronal diameter) could be predictors. The LRPNs with a MIAD ≥ 6.1 mm were considered positive. If the mean SUV ≥ 2.6 or if the maximal coronal diameter ≥ 25 mm and maximal axial diameter ≥ 8 mm, the nodes with MIAD < 6.1 mm should be considered as positive 23. Otherwise, they were negative (Fig. 2).

### Statistical Analysis

The scatter plots for each parameter for LRPNs for positive and negative nodes were illustrated with Excel 2010 version 14.0.7212.5000 (Microsoft, Redmond, WA). The new approach and MIAD ≥ 6.0 mm were tested to derive the respective accuracy for LRPNs, which were indicated by the receiver operating characteristic curve. For comparison of the difference between the two methods, the significance of the difference between the two areas under the curves (AUC) from both approaches was calculated by the Chi-squared test with the null hypothesis considering the two areas under the curves as equal. The statistical analyses were carried out using SAS 9.4 (Cary, NC, USA).

## Results

**Figure 3:**
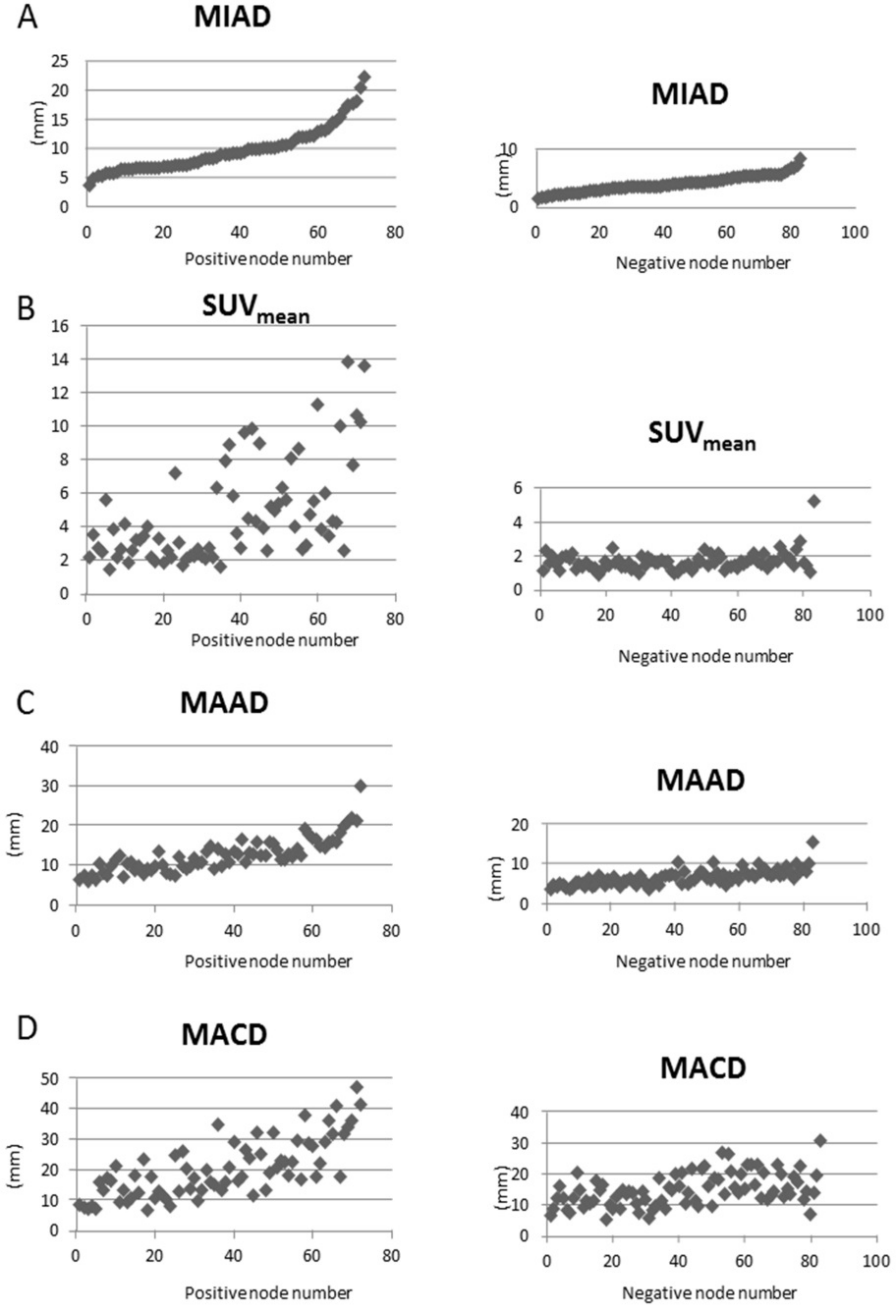
Scatter plots of parameters versus 72 positive nodes on the left side and 83 negative nodes on the right side. **A:** We plotted the minimal axial diameter (MIAD) with the node numbers in abscissa ranked by the MIAD size. The values of other parameters (**B:** mean standard uptake value (SUV_mean)_; **C:** maximal axial diameter (MAAD); **D:** maximal coronal diameter (MACD)) are correspondingly plotted.

**Figure 4:**
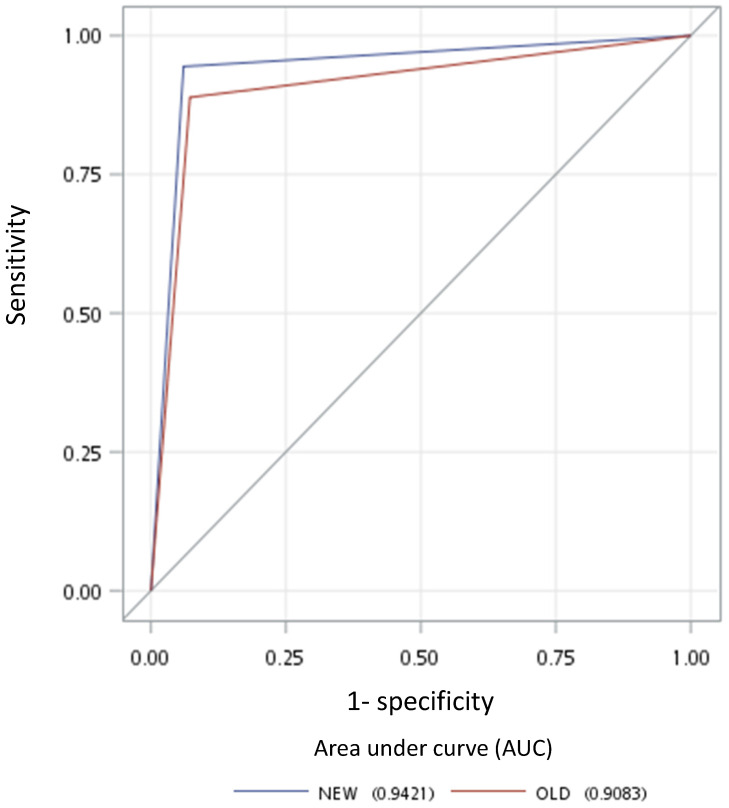
Receiver operating characteristic curves for the new multistage approach (blue line) and old criterion using minimal axial diameter cutoff at ≥6 mm (red line).

A total of 137 patients were initially enrolled for the investigation. Table 1 lists the 67 eligible NPC cases and their clinical characteristics. Among the 155 LRPNs identified from these patients, 72 were positive, and 83 were negative (Fig. 1, with an additional table showing details [see Supplementary Table S1]). All positive and negative nodes for MIAD, maximal axial diameter, maximal coronal diameter, and mean SUV were separately drawn with scatter plots (Fig. 3, with additional tables showing details [see Supplementary Table S1 and S2 respectively]). The results were assessed by using three main qualifications: accuracy: number of correct assessments over the number of all assessments, meant to measure the degree of veracity of a diagnostic test on a condition; specificity: number of true negative assessment over the number of all negative assessment, suggesting how good the test is at identifying negative condition; and sensitivity: number of true positive assessment over the number of all positive assessment, suggesting how good the test is at detecting a disease. The results from traditional MIAD cutoffs at ≥5 mm reached 0.85, 0.74, and 0.97, respectively. The values at ≥6 mm were 0.91 (accuracy), 0.93 (specificity), and 0.89 (sensitivity). The accuracy, specificity, and sensitivity for the multistage approach were all 0.94 (Table 2). Compared with that of the traditional method, the area under the curves (Fig. 4) was significantly greater for the multistage method with a cutoff at ≥6 mm with p=0.023. In our series, 28 LRPNs with a picture of node three or more contiguous confluent, 39 extracapsular extensions, 44 central necrosis, and 40 overt FDG uptake LRPNs were obtained and identified as positive nodes. There is an additional table showing details [see Supplementary Table S3].

## Discussion

Most authors agreed the criterion of LRPNs selected the MIAD ranging from ≥ 5 to ≥ 6 mm in the past two more decades 3-21. As a single parameter, MIAD was proved to be more useful than the maximal axial diameter by Zhang's study 13. In that article, they used the responsiveness and follow-up data to judge LRPNs. This method was reviewed to be robust 14. However, shifting MIAD ≥ 6 mm from ≥5 mm was not accepted worldwide for clinical use due to the survival data and its inconsistency 15, 21. Our findings were consistent with the results showing a higher accuracy to be from MIAD ≥ 6 mm, not from MIAD ≥5 mm (Tables 1 and 2). Therefore we support the single cutoff value of MIAD should be up-shifted to 6 mm.

Specificity and sensitivity can never be enhanced simultaneously by tuning a single parameter, problems will remain unsettled if we do not consider adding other parameters for assessment. Table 2 showed better diagnostic outcomes compared with those from either MIAD ≥ 6 mm or ≥5 mm. The performance improvement of this multistage approach reinforced the benefit of applying our method compared to previous ones 23. Compared with our previous study (Table 3), the current findings were better in all outcome categories. A possible explanation was that the tested nodes might be easier to be judged in this cohort. However, the advantage of both cohorts for the multistage method over a single parameter was the enhancement of specificity and sensitivity, different from shifting a single cutoff value from 5 mm to 6 mm that results in the sacrifice of sensitivity. Under the framework of multistage approach, those nodes with a MIAD smaller than 6.1 mm (after step 1 in Fig. 2), additional inclusion of factors such as nodal mean SUV ≥ 2.6, or maximal axial and coronal diameter ≥ 8 mm and ≥ 25 mm become relevant points to identify false-negatives, as had been mischaracterized by previous methodologies.

PET/CT has been increasingly used in NPC patients because of its sensitivity in detecting distant metastasis (M1) and secondary cancer 22. However, the role of FDG-PET/CT alone in determining the involvement of LRPNs is rarely appreciated 24, 26. As a component of the multistage method, we find the overall accuracy of diagnosis can still be enhanced remarkably by the evaluation of mean SVU. Since LRPN is a special part of the NPC nodal region, our special type of diagnostic criteria demonstrates practical guidance on how the supplementing role of PET/CT can be played, as suggested in the 8^th^ edition of the AJCC staging system. This finding is suggested to be included in the supporting data for NPC nodal staging system in the next version.

Overall in our 155 LRPNs, there were five nodes with different outcomes judged from two methods (Table 4). All nodes with different results showed the new method to be correct. The improved performance of the new method over the traditional method was proved to be statistically different in the AUC in the receiver operating characteristic space with p = 0.023 (Fig. 4). The AUC is equal to the probability that a method will rank a randomly chosen positive instance higher than a randomly chosen negative one 27. Therefore, the AUC, the surrogate of the accuracy of these two methods, is suitable for comparing their difference.

The improvement of accuracy from the multistage approach ranged from 3~4% and 6~10% in comparison with single factor cut at 6 mm and 5 mm, respectively (Tables 2 and 3). This improvement is crucial for specialists dealing with newly diagnosed NPC patients. Reduced sensitivity loss can lower the fear of missing tumor while RT is planning. The involvement of RPLN implies N1 and stage II at least 22. Chemotherapy is favored to be added to RT due to its association with a higher rate of M1 15. A Higher dose of RT to LRPNs could lead to detrimental sequelae such as aspiration, vessel stenosis even stroke, trismus, and cranial cervical nerve injury due to location proximity 28-30. The miseries from these complications usually are chronic since cancer treatment often leads to long-term survival in NPC patients. A higher RT dose is suggested for positive nodes and vice versa, either over- and under-irradiation can be a serious result. The accurate diagnosis of an LRPN is very important and means that we should jump at any chance to improve it.

The limitations of this study included the lack of radiologic-histopathologic correlation data. The feasibility of histological confirmation before ongoing chemotherapy or RT for LRPNs in newly diagnosed NPC is still lacking, although several biopsy methods had been reported; still, several procedures could be safe 31-35. However, these procedures are only applied to the recurrent LRPNs and not for new cases before RT. The typical images of our nodes agreed with the positive nodes by RT response follow-up data. Using the criteria for assessing LRPNs with RT response remains the robust standard methodology 14. Second, the cases in our series were limited. Thus, different cohorts must be recruited to determine whether consistent results could be met. We did not have PET/magnetic resonance, which could aid in the evaluation of the mean SUV data in small nodes such as LRPNs 36-38. We also did not consider other parameters, such as diffusion-weighted imaging in MRI and the effect of Epstein-Barr virus DNA serum titer 39, 40. These parameters could be applied in future studies. Finally, the obtained accuracy improvement from adopting the multistage method was relatively small but statistically significant. Given the lack of feasibility of resorting to the final judgment of an LRPN to histological confirmation as a backup in clinical treatment decision making for newly diagnosed NPC patients, clinicians are encouraged to apply this new method despite its long period of operation.

## Conclusion

As a single cutoff value for MIAD, 6 mm is better than 5 mm. This study supported the advantage of the multistage approach. LRPNs in NPC patients with a MIAD ≥ 6.1 mm should determine as positive. Among nodes with a MIAD < 6.1 mm, if the mean SUV ≥ 2.6 or maximal coronal diameter ≥ 25 mm and maximal axial diameter ≥ 8 mm, the nodes should also be considered as positive.

## Supplementary Material

Supplementary tables.

Supplementary figures.

## Figures and Tables

**Table 1:** Clinical characteristics of 67 eligible nasopharyngeal carcinoma patients

Characteristic	Number of patients (percentage)
**Age (years)**	
Age < 40	15 (22)
Age ≥ 40	52 (78)
**Sex**	
Male	46 (69)
Female	21 (31)
**World Health Organization pathologic feature**	
Keratinizing squamous cell carcinoma	0 (0)
Non-keratinizing carcinoma (not otherwise specified)	9 (13 )
Non-keratinizing differentiated carcinoma	23 (34)
Non-keratinizing undifferentiated carcinoma	34 (51)
Not applicable	1 (1 )
**American Joint Committee on Cancer 2010 stage**	
I	3 (4)
II	11 (16)
III	23 (34)
IV	30 (45)

**Table 2:** Accuracy results of the multistage and conventional criteria for 155 lateral retropharyngeal nodes in our patients

Criteria for 155 nodes	Accuracy	Specificity	Sensitivity	Positive predictive value	Negative predictive value
MIAD ≥ 5.0 mm	0.845	0.735	0.972	0.761	0.968
MIAD ≥ 6.0 mm	0.910	0.928	0.889	0.914	0.906
Multi-stage method	0.942	0.940	0.944	0.932	0.951

MIAD: minimal axial diameter.

**Table 3:** Results of multistage and two conventional criteria for 410 nodes in our previous report 23

Criteria for 410 nodes	Accuracy	Specificity	Sensitivity	Positive predictive value	Negative predictive value
MIAD ≥ 5.0 mm	0.846	0.779	0.910	0.814	0.891
MIAD ≥ 6.0 mm	0.890	0.930	0.853	0.928	0.856
Multi-stage method	0.905	0.950	0.863	0.948	0.867

MIAD: minimal axial diameter.

**Table 4:** Predicted conditions of the multistage and conventional criteria for 155 lateral retropharyngeal nodes in our patients

	MIAD ≥ 6.0 mm		Total
	Negative	Positive	
**Multi-stage method**			
Negative	81	1	82
Positive	4	69	73
Total	85	70	155

MIAD: minimal axial diameter.
