# Supplementary material for: Verification of the Efficacy of New Diagnostic Criteria for Retropharyngeal Nodes in a Cohort of Nasopharyngeal Carcinoma Patients
Source: Int J Med Sci. 2021 Aug 13;18(15):3463–9. doi: 10.7150/ijms.58375 (PMC8436115; doi:10.7150/ijms.58375)
Supplement: Supplementary file 1 — Supplementary tables. [file ijmsv18p3463s1.pdf]

Supplementary materials

Table S1. Data complementary to flowchart outlining of the follow-up MRI results of 155 LRPNs in 67 patients in Figure 1 of the main text

Node number	MACD	MAAD	MIAD	RTed MACD	RTed MAAD	RTed MIAD	Ratio	Real nature
1	16	15	8.5	10.1	6.2	2.6	0.261	1
2	9.3	10.3	6.5	5.9	1.9	1.4	0.117	1
3	6.4	3.6	1.4	4.3	3.2	1.6	0.597	0
4	14.9	5.3	2.2	11.8	6.6	3.1	0.986	0
5	16.1	5	1.8	10.6	8.3	4.1	1.093	0
6	21.8	6.7	4	21.4	6.8	3.1	0.996	0
7	41.7	30.1	22.1	25.1	11.9	11.8	0.238	1
8	9.9	6.2	2.8	8.6	5.5	2.2	0.771	0
9	14.1	6	4.5	9.1	5.4	4.4	0.581	0
10	16.2	10.6	5.7	9.3	3.6	2.8	0.195	1
11	13.7	5.8	3.1	12.4	4.5	4	0.702	0
12	15.1	7	5.5	12.7	7.5	5.2	0.901	0
13	16.7	6.4	2.6	17.7	6.6	2.3	1.093	0
14	9	5.2	2.2	6.4	4.6	3.6	0.629	0
15	14.4	7	3.3	13.5	6.7	3	0.897	0
16	5.3	4.7	2.7	5.6	3	2.2	0.674	0
17	9.6	5	3.4	6	4.2	2.2	0.525	0
18	32.2	15.9	10	12.5	5	2.6	0.122	1
19	12.9	13.6	6.9	4.4	5.5	2.2	0.138	1
20	16.1	7	3.6	10	5.9	1.6	0.524	0
21	12.6	6.7	2.9	10.7	7	3	0.887	0
22	11.8	5.8	3.3	11.9	5.4	3.2	0.939	0
23	16.6	9.9	6.4	5.5	5	3	0.167	1
24	20.8	13.9	10.4	14.1	5.1	1.9	0.249	1
25	12.4	6.9	5.2	10.4	6.3	2.7	0.766	0
26	17.9	8.6	6.6	14.1	4.9	1.2	0.449	1
27	21.5	11.4	6.4	18.2	6.5	1.9	0.483	1
28	14.1	8	3.8	13.4	4.6	3.1	0.546	0
29	22.5	12	11.8	13.4	4.5	1.8	0.223	1
30	8.2	7.6	7.1	6.4	4.3	1.6	0.442	1
31	23	7.4	5.1	23	7.8	4.3	1.054	0
32	18.5	6	3.4	18.5	7	4.5	1.167	0
33	17.5	11.9	8	13.3	7.6	3.8	0.485	1
34	7.6	3.7	2.1	7.5	4	2.4	1.067	0
35	11.3	4.3	2.3	7.4	4.5	1.7	0.685	0

36	18.4	10.5	4.2	18.6	6.7	4.3	0.645	0
37	17.2	8	5.6	15.8	8	5.2	0.919	0
38	13.2	8.8	5.3	13.6	6.8	4	0.796	0
39	15	9	8.8	10.6	4	2.53	0.314	1
40	29.6	14	11.9	13.1	7.1	2.8	0.224	1
41	20.5	10.4	3.6	18.4	7.5	3.6	0.647	0
42	14.3	5.7	3.1	8.8	6.3	2.5	0.680	0
43	25.4	12.5	10	18.2	6.5	3.9	0.373	1
44	21.8	5.1	3.9	21.1	6.1	4.6	1.158	0
45	10.8	10.8	6.5	7	3.6	2.3	0.216	1
46	14.5	7.6	5.3	17.5	6.6	4.6	1.048	0
47	18.1	12.8	11.5	10.4	5.7	5.2	0.256	1
48	20.8	4.7	4.4	18.3	3.7	1.8	0.693	0
49	34.1	20.8	17.5	16.7	4.9	2.7	0.115	1
50	19.5	10.2	7.1	17.4	7.8	5.9	0.682	0
51	7.4	6	5.1	4.6	3.8	2.4	0.394	1
52	18	18.3	16.4	6.4	3.4	2.4	0.066	1
53	11	9.7	6.7	9	5.3	1.9	0.447	1
54	11.3	4.9	3.4	10	4.9	2.8	0.885	0
55	8.2	7.5	5.2	6.9	4.2	3.9	0.471	1
56	22.3	14.8	13.1	18.4	8.9	7.6	0.496	1
57	10.5	5.4	3.2	5.9	6.6	2.2	0.687	0
58	19.8	7.6	5.4	16.2	6.5	4.6	0.700	0
59	36.1	15.9	14.5	15	4.1	3.9	0.107	1
60	17	12.5	11.9	10.4	6	3.7	0.294	1
61	9.6	6.5	4.1	8.1	4.3	2	0.558	0
62	23	7.2	5.3	21.7	7.1	4.1	0.930	0
63	38.1	19.1	12.1	19.3	10.1	6.1	0.268	1
64	18.2	8.9	6.5	8.7	5.4	3	0.290	1
65	20.6	9.2	7.5	13.9	5.5	3.7	0.403	1
66	8.3	4	1.9	10.8	2.8	2.4	0.911	0
67	17.8	16.6	9.7	5.7	5.4	3.8	0.104	1
68	8.9	5.5	2.8	9.7	5.5	4.1	1.090	0
69	22.5	11.5	10.8	7.1	6.3	5.7	0.173	1
70	15.6	6.8	4.4	12	5.4	1.8	0.611	0
71	13.5	5.6	4.3	7.9	6.3	3.2	0.658	0
72	9.7	12.4	6.4	7.7	7.8	6	0.499	1
73	6.9	8.9	6.6	7.2	6.1	5.4	0.715	0
74	16.5	12.7	9.3	8.3	5.6	4	0.222	1
75	12.4	9.9	6.5	9.5	6.1	2.6	0.472	1

76	13.5	7.4	5.5	12.6	5.4	1.7	0.681	0
77	36.1	22	18.1	18.9	6.7	5.2	0.159	1
78	11.5	4.4	2.2	9.4	4.6	2.6	0.855	0
79	10.6	6.3	2.2	9.6	7	2.5	1.006	0
80	12.3	4	2.1	12.1	3.5	2	0.861	0
81	20.3	5.5	2.1	19.2	4.9	1.8	0.843	0
82	12.1	4.3	1.6	11.7	4.3	2.8	0.967	0
83	8.7	5.5	3	8.2	5.8	1.1	0.994	0
84	14.6	4.5	3	14.4	4.8	3	1.052	0
85	8.6	4.9	1.5	8.7	4.5	1.8	0.929	0
86	15.8	7.1	3.5	11.9	5.9	3.1	0.626	0
87	32.4	15.4	10.2	13.4	6.4	2.5	0.172	1
88	20.8	10.7	9.1	14.8	6.2	3.4	0.412	1
89	18	16.7	13	8.4	3.5	2.3	0.098	1
90	20.6	10	5.2	15.9	6.7	3.3	0.517	0
91	13.4	10.8	8.2	8.9	5.3	5	0.326	1
92	18	4.7	2.5	11.9	4.7	2.7	0.661	0
93	13.4	12.5	10.1	7.7	5.2	3.6	0.239	1
94	5.6	5	3.3	5.3	5	3	0.946	0
95	15	7.1	2.5	15.2	4.7	2.7	0.671	0
96	12.4	4.6	1.8	9.7	5	2.4	0.850	0
97	27	7.8	4.2	23	7.9	4.2	0.863	0
98	22.5	8.3	4	22	7.5	3.5	0.884	0
99	26.5	10.9	9.7	18.3	5.8	3.9	0.367	1
100	13.4	7.9	5.7	11.1	4.4	2.2	0.461	1
101	29.3	13.5	9.2	15.8	7	4.1	0.280	1
102	11.7	8.2	5.2	12.4	6.1	2.6	0.788	0
103	22.5	6.4	5.6	22.5	4.9	3.8	0.766	0
104	19.3	9.5	5.5	14.5	7.7	2.1	0.609	0
105	17.6	7.3	5.8	11.8	3.7	2.6	0.340	1
106	25.1	7.5	7.1	11.4	5.1	3.9	0.309	1
107	23.3	11.5	10.5	11.8	4.3	2.9	0.189	1
108	13.8	6.5	3.1	13.8	5.6	3.1	0.862	0
109	11.4	5.6	3.9	8.4	4.8	3.1	0.632	0
110	23.2	8.1	5	17.9	9.9	4.9	0.943	0
111	10.5	5.2	3.7	7.7	5.2	2.4	0.733	0
112	23.5	7.8	6.5	17.2	3.4	1.9	0.319	1
113	19.2	15.7	10.1	20.6	7.1	4.4	0.485	1
114	9.5	6.1	3.9	9.1	3.9	2.8	0.612	0
115	16.4	7	5	11.3	7.6	2.6	0.748	0

116	23.8	13.1	9.7	11	5.6	4.6	0.198	1
117	20	7.1	4.6	25.8	4.9	3.5	0.890	0
118	12.7	8.8	5.4	14.5	7.2	39	0.934	0
119	16.2	12.7	9	3.9	5.8	3	0.110	1
120	7.5	4.7	3.2	7.5	2.5	2	0.532	0
121	15.2	6	4.7	12.3	4.8	2.7	0.647	0
122	13.8	8	6.7	15.6	7.2	5.4	1.017	0
123	14.8	7.2	3.5	10.6	5.7	3.4	0.567	0
124	41	16	15.3	17.2	5.2	3.7	0.136	1
125	47.1	21.2	20.4	14.2	7.7	4.8	0.110	1
126	11.7	12.7	9.9	5.8	5.7	4.2	0.222	1
127	13.7	9.8	7.6	9.5	4.9	5.7	0.347	1
128	30.9	15.7	8.3	24.3	11.3	6.4	0.566	0
129	14.2	8.3	6.2	13.9	5.3	3.4	0.625	0
130	20.2	13.4	8.2	14.3	6.6	2.6	0.349	1
131	29.1	14.4	13.5	18.3	9.1	3.7	0.397	1
132	35.2	14.3	8.8	9.9	6.1	2.2	0.120	1
133	11.6	10.1	6	8	9.6	5.3	0.656	0
134	26.7	7.4	4.3	22.2	8.1	6.4	0.910	0
135	23.1	9.8	4.8	20.5	10.2	4.6	0.924	0
136	11.6	10	6.9	9.2	4.9	4	0.389	1
137	16.3	7.7	4	11.5	8.7	4.3	0.797	0
138	13.3	9.8	8.9	9.2	3.2	2.6	0.226	1
139	7.6	7.3	4.7	4.8	3.6	2.4	0.311	1
140	10.5	8.1	7	4.4	4	2.2	0.207	1
141	8.6	6.7	3.4	7.4	4.5	3.6	0.578	0
142	6.8	9	6.5	4.3	4.8	2.6	0.337	1
143	20	7.5	3.5	22.8	7.1	4.1	1.079	0
144	18.6	6.2	4.1	19.5	6.2	3.3	1.048	0
145	8.4	3.8	3.3	7.4	4.4	4.3	1.020	0
146	10.1	10.4	8.1	7.4	5.7	3.8	0.402	1
147	7.2	6.2	5.6	4.6	3.9	2.7	0.402	1
148	28.9	17.9	12.1	15.5	4.9	3.3	0.147	1
149	32	16.1	14.7	13.8	6.7	4.7	0.179	1
150	32	19.8	17.3	15.8	6.9	3.3	0.172	1
151	27.9	16.8	12.7	13.8	6.2	3.4	0.183	1
152	8.8	6.3	3.5	7	3.5	3.3	0.442	1
153	13.3	7.1	6.4	11.9	2.7	2.7	0.340	1
154	26.4	9.7	7.2	14.1	8.4	4.7	0.463	1
155	13.1	12	7.1	11.2	6.9	4.3	0.492	1

Total	Positive	72
	Negative	83

MRI: Magnetic resonance imaging; LRPNs: lateral retropharyngeal nodes; RTed: irradiated; MAAD: maximal axial diameter; MACD: maximal coronal diameter; MIAD: minimal axial diameter. The unit for the diameter is in mm. Ratio indicates the value of product of two long axes in MRI after radiation therapy divided by that before radiation therapy. Negative node is defined as the ration ranged from 1.25 to 0.5 without progress after follow up periods at least for 6 months. Real nature of the positive nodes are represented by "1" and the negatives, by "0".

Table S2. Original minimal axial diameter data (MIAD) of 155 nodes and their results tested by the conventional criterion of MIAD ≥ 6.0 mm

Node number	Real nature	MIAD	Test by MIAD ≥ 6.0	TP	TN	FP	FN
1	1	8.5	1	1	0	0	0
2	1	6.5	1	1	0	0	0
3	0	1.4	0	0	1	0	0
4	0	2.2	0	0	1	0	0
5	0	1.8	0	0	1	0	0
6	0	4	0	0	1	0	0
7	1	22.1	1	1	0	0	0
8	0	2.8	0	0	1	0	0
9	0	4.5	0	0	1	0	0
10	1	5.7	0	0	0	0	1
11	0	3.1	0	0	1	0	0
12	0	5.5	0	0	1	0	0
13	0	2.6	0	0	1	0	0
14	0	2.2	0	0	1	0	0
15	0	3.3	0	0	1	0	0
16	0	2.7	0	0	1	0	0
17	0	3.4	0	0	1	0	0
18	1	10	1	1	0	0	0
19	1	6.9	1	1	0	0	0
20	0	3.6	0	0	1	0	0
21	0	2.9	0	0	1	0	0
22	0	3.3	0	0	1	0	0
23	1	6.4	1	1	0	0	0
24	1	10.4	1	1	0	0	0
25	0	5.2	0	0	1	0	0
26	1	6.6	1	1	0	0	0
27	1	6.4	1	1	0	0	0
28	0	3.8	0	0	1	0	0
29	1	11.8	1	1	0	0	0
30	1	7.1	1	1	0	0	0
31	0	5.1	0	0	1	0	0
32	0	3.4	0	0	1	0	0
33	1	8	1	1	0	0	0
34	0	2.1	0	0	1	0	0
35	0	2.3	0	0	1	0	0
36	0	4.2	0	0	1	0	0

37	0	5.6	0	0	1	0	0
38	0	5.3	0	0	1	0	0
39	1	8.8	1	1	0	0	0
40	1	11.9	1	1	0	0	0
41	0	3.6	0	0	1	0	0
42	0	3.1	0	0	1	0	0
43	1	10	1	1	0	0	0
44	0	3.9	0	0	1	0	0
45	1	6.5	1	1	0	0	0
46	0	5.3	0	0	1	0	0
47	1	11.5	1	1	0	0	0
48	0	4.4	0	0	1	0	0
49	1	17.5	1	1	0	0	0
50	0	7.1	1	0	0	1	0
51	1	5.1	0	0	0	0	1
52	1	16.4	1	1	0	0	0
53	1	6.7	1	1	0	0	0
54	0	3.4	0	0	1	0	0
55	1	5.2	0	0	0	0	1
56	1	13.1	1	1	0	0	0
57	0	3.2	0	0	1	0	0
58	0	5.4	0	0	1	0	0
59	1	14.5	1	1	0	0	0
60	1	11.9	1	1	0	0	0
61	0	4.1	0	0	1	0	0
62	0	5.3	0	0	1	0	0
63	1	12.1	1	1	0	0	0
64	1	6.5	1	1	0	0	0
65	1	7.5	1	1	0	0	0
66	0	1.9	0	0	1	0	0
67	1	9.7	1	1	0	0	0
68	0	2.8	0	0	1	0	0
69	1	10.8	1	1	0	0	0
70	0	4.4	0	0	1	0	0
71	0	4.3	0	0	1	0	0
72	1	6.4	1	1	0	0	0
73	0	6.6	1	0	0	1	0
74	1	9.3	1	1	0	0	0
75	1	6.5	1	1	0	0	0
76	0	5.5	0	0	1	0	0

77	1	18.1	1	1	0	0	0
78	0	2.2	0	0	1	0	0
79	0	2.2	0	0	1	0	0
80	0	2.1	0	0	1	0	0
81	0	2.1	0	0	1	0	0
82	0	1.6	0	0	1	0	0
83	0	3	0	0	1	0	0
84	0	3	0	0	1	0	0
85	0	1.5	0	0	1	0	0
86	0	3.5	0	0	1	0	0
87	1	10.2	1	1	0	0	0
88	1	9.1	1	1	0	0	0
89	1	13	1	1	0	0	0
90	0	5.2	0	0	1	0	0
91	1	8.2	1	1	0	0	0
92	0	2.5	0	0	1	0	0
93	1	10.1	1	1	0	0	0
94	0	3.3	0	0	1	0	0
95	0	2.5	0	0	1	0	0
96	0	1.8	0	0	1	0	0
97	0	4.2	0	0	1	0	0
98	0	4	0	0	1	0	0
99	1	9.7	1	1	0	0	0
100	1	5.7	0	0	0	0	1
101	1	9.2	1	1	0	0	0
102	0	5.2	0	0	1	0	0
103	0	5.6	0	0	1	0	0
104	0	5.5	0	0	1	0	0
105	1	5.8	0	0	0	0	1
106	1	7.1	1	1	0	0	0
107	1	10.5	1	1	0	0	0
108	0	3.1	0	0	1	0	0
109	0	3.9	0	0	1	0	0
110	0	5	0	0	1	0	0
111	0	3.7	0	0	1	0	0
112	1	6.5	1	1	0	0	0
113	1	10.1	1	1	0	0	0
114	0	3.9	0	0	1	0	0
115	0	5	0	0	1	0	0
116	1	9.7	1	1	0	0	0

117	0	4.6	0	0	1	0	0
118	0	5.4	0	0	1	0	0
119	1	9	1	1	0	0	0
120	0	3.2	0	0	1	0	0
121	0	4.7	0	0	1	0	0
122	0	6.7	1	0	0	1	0
123	0	3.5	0	0	1	0	0
124	1	15.3	1	1	0	0	0
125	1	20.4	1	1	0	0	0
126	1	9.9	1	1	0	0	0
127	1	7.6	1	1	0	0	0
128	0	8.3	1	0	0	1	0
129	0	6.2	1	0	0	1	0
130	1	8.2	1	1	0	0	0
131	1	13.5	1	1	0	0	0
132	1	8.8	1	1	0	0	0
133	0	6	1	0	0	1	0
134	0	4.3	0	0	1	0	0
135	0	4.8	0	0	1	0	0
136	1	6.9	1	1	0	0	0
137	0	4	0	0	1	0	0
138	1	8.9	1	1	0	0	0
139	1	4.7	0	0	0	0	1
140	1	7	1	1	0	0	0
141	0	3.4	0	0	1	0	0
142	1	6.5	1	1	0	0	0
143	0	3.5	0	0	1	0	0
144	0	4.1	0	0	1	0	0
145	0	3.3	0	0	1	0	0
146	1	8.1	1	1	0	0	0
147	1	5.6	0	0	0	0	1
148	1	12.1	1	1	0	0	0
149	1	14.7	1	1	0	0	0
150	1	17.3	1	1	0	0	0
151	1	12.7	1	1	0	0	0
152	1	3.5	0	0	0	0	1
153	1	6.4	1	1	0	0	0
154	1	7.2	1	1	0	0	0
155	1	7.1	1	1	0	0	0
Total “1”				64	77	6	8

ACC	SPE	SEN	PPV	NPV
0.910	0.928	0.889	0.914	0.906

MIAD: minimal axial diameter; TP: true positive; TN: true negative; FP: false positive; FN: false negative; ACC: accuracy; SPE: specificity = SEN: sensitivity; PPV: Positive prediction value; NPV: Negative prediction value. The unit for the diameter is in mm. All “1” in table represents positive while “0”, the negative.

Table S3. Original parameter values of 155 nodes and their results tested by the multistage (new) approach

Node number	Real nature	MIAD	MAAD	MACD	SUV_{mean}	Test by new	TP	TN	FP	FN
1	1	8.5	15	16	6.4	1	1	0	0	0
2	1	6.5	10.3	9.3	3.24	1	1	0	0	0
3	0	1.4	3.6	6.4	1.14	0	0	1	0	0
4	0	2.2	5.3	14.9	2.21	0	0	1	0	0
5	0	1.8	5	16.1	2	0	0	1	0	0
6	0	4	6.7	21.8	1.36	0	0	1	0	0
7	1	22.1	30.1	41.7	13.68	1	1	0	0	0
8	0	2.8	6.2	9.9	1.22	0	0	1	0	0
9	0	4.5	6	14.1	1.37	0	0	1	0	0
10	1	5.7	10.6	16.2	1.52	0	0	0	0	1
11	0	3.1	5.8	13.7	1.78	0	0	1	0	0
12	0	5.5	7	15.1	2.56	0	0	1	0	0
13	0	2.6	6.4	16.7	1.33	0	0	1	0	0
14	0	2.2	5.2	9	1.22	0	0	1	0	0
15	0	3.3	7	14.4	1.34	0	0	1	0	0
16	0	2.7	4.7	5.3	0.91	0	0	1	0	0
17	0	3.4	5	9.6	1.95	0	0	1	0	0
18	1	10	15.9	32.2	4.01	1	1	0	0	0
19	1	6.9	13.6	12.9	2.6	1	1	0	0	0
20	0	3.6	7	16.1	1.12	0	0	1	0	0
21	0	2.9	6.7	12.6	1.44	0	0	1	0	0
22	0	3.3	5.8	11.8	0.98	0	0	1	0	0
23	1	6.4	9.9	16.6	2.71	1	1	0	0	0
24	1	10.4	13.9	20.8	6.39	1	1	0	0	0
25	0	5.2	6.9	12.4	2.19	0	0	1	0	0
26	1	6.6	8.6	17.9	3.3	1	1	0	0	0
27	1	6.4	11.4	21.5	4.25	1	1	0	0	0
28	0	3.8	8	14.1	1.43	0	0	1	0	0
29	1	11.8	12	22.5	8.68	1	1	0	0	0
30	1	7.1	7.6	8.2	3.12	1	1	0	0	0
31	0	5.1	7.4	23	1.88	0	0	1	0	0
32	0	3.4	6	18.5	1.82	0	0	1	0	0
33	1	8	11.9	17.5	2.3	1	1	0	0	0
34	0	2.1	3.7	7.6	1.95	0	0	1	0	0
35	0	2.3	4.3	11.3	1.6	0	0	1	0	0
36	0	4.2	10.5	18.4	2.17	0	0	1	0	0
37	0	5.6	8	17.2	1.9	0	0	1	0	0

38	0	5.3	8.8	13.2	2.15	0	0	1	0	0
39	1	8.8	9	15	1.64	1	1	0	0	0
40	1	11.9	14	29.6	2.69	1	1	0	0	0
41	0	3.6	10.4	20.5	1.03	0	0	1	0	0
42	0	3.1	5.7	14.3	1.36	0	0	1	0	0
43	1	10	12.5	25.4	2.6	1	1	0	0	0
44	0	3.9	5.1	21.8	1.36	0	0	1	0	0
45	1	6.5	10.8	10.8	3.23	1	1	0	0	0
46	0	5.3	7.6	14.5	1.35	0	0	1	0	0
47	1	11.5	12.8	18.1	4.06	1	1	0	0	0
48	0	4.4	4.7	20.8	1.14	0	0	1	0	0
49	1	17.5	20.8	34.1	7.71	1	1	0	0	0
50	0	7.1	10.2	19.5	1.05	1	0	0	1	0
51	1	5.1	6	7.4	2.81	1	1	0	0	0
52	1	16.4	18.3	18	2.62	1	1	0	0	0
53	1	6.7	9.7	11	1.89	1	1	0	0	0
54	0	3.4	4.9	11.3	1.62	0	0	1	0	0
55	1	5.2	7.5	8.2	2.5	0	0	0	0	1
56	1	13.1	14.8	22.3	6.08	1	1	0	0	0
57	0	3.2	5.4	10.5	1.66	0	0	1	0	0
58	0	5.4	7.6	19.8	1.72	0	0	1	0	0
59	1	14.5	15.9	36.1	4.41	1	1	0	0	0
60	1	11.9	12.5	17	2.91	1	1	0	0	0
61	0	4.1	6.5	9.6	2.42	0	0	1	0	0
62	0	5.3	7.2	23	1.73	0	0	1	0	0
63	1	12.1	19.1	38.1	4.8	1	1	0	0	0
64	1	6.5	8.9	18.2	3.53	1	1	0	0	0
65	1	7.5	9.2	20.6	2.3	1	1	0	0	0
66	0	1.9	4	8.3	1.15	0	0	1	0	0
67	1	9.7	16.6	17.8	4.5	1	1	0	0	0
68	0	2.8	5.5	8.9	1.71	0	0	1	0	0
69	1	10.8	11.5	22.5	8.13	1	1	0	0	0
70	0	4.4	6.8	15.6	1.42	0	0	1	0	0
71	0	4.3	5.6	13.5	2.15	0	0	1	0	0
72	1	6.4	12.4	9.7	1.87	1	1	0	0	0
73	0	6.6	8.9	6.9	1.6	1	0	0	1	0
74	1	9.3	12.7	16.5	9.69	1	1	0	0	0
75	1	6.5	9.9	12.4	4.05	1	1	0	0	0
76	0	5.5	7.4	13.5	2.17	0	0	1	0	0
77	1	18.1	22	36.1	10.67	1	1	0	0	0

78	0	2.2	4.4	11.5	1.54	0	0	1	0	0
79	0	2.2	6.3	10.6	1.38	0	0	1	0	0
80	0	2.1	4	12.3	2	0	0	1	0	0
81	0	2.1	5.5	20.3	1.85	0	0	1	0	0
82	0	1.6	4.3	12.1	1.61	0	0	1	0	0
83	0	3	5.5	8.7	2.48	0	0	1	0	0
84	0	3	4.5	14.6	1.6	0	0	1	0	0
85	0	1.5	4.9	8.6	2.36	0	0	1	0	0
86	0	3.5	7.1	15.8	1.81	0	0	1	0	0
87	1	10.2	15.4	32.4	5.39	1	1	0	0	0
88	1	9.1	10.7	20.8	3.65	1	1	0	0	0
89	1	13	16.7	18	3.89	1	1	0	0	0
90	0	5.2	10	20.6	1.68	0	0	1	0	0
91	1	8.2	10.8	13.4	2.74	1	1	0	0	0
92	0	2.5	4.7	18	1.49	0	0	1	0	0
93	1	10.1	12.5	13.4	5.22	1	1	0	0	0
94	0	3.3	5	5.6	2.02	0	0	1	0	0
95	0	2.5	7.1	15	1.31	0	0	1	0	0
96	0	1.8	4.6	12.4	1.55	0	0	1	0	0
97	0	4.2	7.8	27	1.6	0	0	1	0	0
98	0	4	8.3	22.5	1.87	0	0	1	0	0
99	1	9.7	10.9	26.5	9.91	1	1	0	0	0
100	1	5.7	7.9	13.4	3.91	1	1	0	0	0
101	1	9.2	13.5	29.3	2.76	1	1	0	0	0
102	0	5.2	8.2	11.7	1.57	0	0	1	0	0
103	0	5.6	6.4	22.5	1.48	0	0	1	0	0
104	0	5.5	9.5	19.3	1.85	0	0	1	0	0
105	1	5.8	7.3	17.6	2.22	0	0	0	0	1
106	1	7.1	7.5	25.1	1.77	1	1	0	0	0
107	1	10.5	11.5	23.3	5.69	1	1	0	0	0
108	0	3.1	6.5	13.8	1.38	0	0	1	0	0
109	0	3.9	5.6	11.4	1.53	0	0	1	0	0
110	0	5	8.1	23.2	1.55	0	0	1	0	0
111	0	3.7	5.2	10.5	1.1	0	0	1	0	0
112	1	6.5	7.8	23.5	2.2	1	1	0	0	0
113	1	10.1	15.7	19.2	4.98	1	1	0	0	0
114	0	3.9	6.1	9.5	1.13	0	0	1	0	0
115	0	5	7	16.4	1.73	0	0	1	0	0
116	1	9.7	13.1	23.8	4.41	1	1	0	0	0
117	0	4.6	7.1	20	1.48	0	0	1	0	0

118	0	5.4	8.8	12.7	1.7	0	0	1	0	0
119	1	9	12.7	16.2	5.89	1	1	0	0	0
120	0	3.2	4.7	7.5	1.26	0	0	1	0	0
121	0	4.7	6	15.2	1.34	0	0	1	0	0
122	0	6.7	8	13.8	1.48	1	0	0	1	0
123	0	3.5	7.2	14.8	1.61	0	0	1	0	0
124	1	15.3	16	41	10.09	1	1	0	0	0
125	1	20.4	21.2	47.1	10.32	1	1	0	0	0
126	1	9.9	12.7	11.7	9..00	1	1	0	0	0
127	1	7.6	9.8	13.7	2.71	1	1	0	0	0
128	0	8.3	15.7	30.9	5.29	1	0	0	1	0
129	0	6.2	8.3	14.2	2.88	1	0	0	1	0
130	1	8.2	13.4	20.2	2.2	1	1	0	0	0
131	1	13.5	14.4	29.1	3.49	1	1	0	0	0
132	1	8.8	14.3	35.2	7.99	1	1	0	0	0
133	0	6	10.1	11.6	2.45	0	0	1	0	0
134	0	4.3	7.4	26.7	1.96	0	0	1	0	0
135	0	4.8	9.8	23.1	1.68	0	0	1	0	0
136	1	6.9	10	11.6	2.23	1	1	0	0	0
137	0	4	7.7	16.3	1.71	0	0	1	0	0
138	1	8.9	9.8	13.3	8.92	1	1	0	0	0
139	1	4.7	7.3	7.6	3.57	1	1	0	0	0
140	1	7	8.1	10.5	7.28	1	1	0	0	0
141	0	3.4	6.7	8.6	1.62	0	0	1	0	0
142	1	6.5	9	6.8	1.98	1	1	0	0	0
143	0	3.5	7.5	20	1.7	0	0	1	0	0
144	0	4.1	6.2	18.6	1.49	0	0	1	0	0
145	0	3.3	3.8	8.4	1.48	0	0	1	0	0
146	1	8.1	10.4	10.1	2.11	1	1	0	0	0
147	1	5.6	6.2	7.2	5.69	1	1	0	0	0
148	1	12.1	17.9	28.9	5.56	1	1	0	0	0
149	1	14.7	16.1	32	4.31	1	1	0	0	0
150	1	17.3	19.8	32	13.9	1	1	0	0	0
151	1	12.7	16.8	27.9	11.38	1	1	0	0	0
152	1	3.5	6.3	8.8	2.18	0	0	0	0	1
153	1	6.4	7.1	13.3	2.6	1	1	0	0	0
154	1	7.2	9.7	26.4	2.27	1	1	0	0	0
155	1	7.1	12	13.1	2.13	1	1	0	0	0
Total “1”							68	78	5	4
ACC	SPE	SEN	PPV	NPV						

0.942	0.940	0.944	0.932	0.951

MIAD: minimal axial diameter; SUV_{mean}: mean standard uptake value; TP: true positive; TN: true negative; FP: false positive; FN: false negative; ACC: accuracy; SPE: specificity; SEN: sensitivity; PPV: Positive prediction value; NPV: Negative prediction value. The unit for the diameter is in mm. All “1” in table represents positive while “0”, the negative.

Table S4. The distribution of characteristics of apparent positive nodes in MRI and overt avid in FDG-PET corresponding to the real nature of nodes decided by radiotherapy response (ratio)

Node number	ECE	CN	Grouping	Ratio	Real nature	SUV_{mean}	Overt FDG avid
1	1	1	1	0.261	1	6.4	1
2	1	1	1	0.117	1	3.24	0
3	0	0	0	0.597	0	1.14	0
4	0	0	0	0.986	0	2.21	0
5	0	0	0	1.093	0	2	0
6	0	0	0	0.996	0	1.36	0
7	1	1	1	0.238	1	13.68	1
8	0	0	0	0.771	0	1.22	0
9	0	0	0	0.581	0	1.37	0
10	0	0	0	0.195	1	1.52	0
11	0	0	0	0.702	0	1.78	0
12	0	0	0	0.901	0	2.56	0
13	0	0	0	1.093	0	1.33	0
14	0	0	0	0.629	0	1.22	0
15	0	0	0	0.897	0	1.34	0
16	0	0	0	0.674	0	0.91	0
17	0	0	0	0.525	0	1.95	0
18	1	1	1	0.122	1	4.01	1
19	1	1	1	0.138	1	2.6	0
20	0	0	0	0.524	0	1.12	0
21	0	0	0	0.887	0	1.44	0
22	0	0	0	0.939	0	0.98	0
23	0	0	0	0.167	1	2.71	0
24	1	1	1	0.249	1	6.39	1
25	0	0	0	0.766	0	2.19	0
26	0	0	0	0.449	1	3.3	0
27	0	0	0	0.483	1	4.25	1
28	0	0	0	0.546	0	1.43	0
29	1	1	1	0.223	1	8.68	1
30	0	1	1	0.442	1	3.12	0
31	0	0	0	1.054	0	1.88	0
32	0	0	0	1.167	0	1.82	0
33	1	0	0	0.485	1	2.3	0
34	0	0	0	1.067	0	1.95	0
35	0	0	0	0.685	0	1.6	0
36	0	0	0	0.645	0	2.17	0

37	0	0	0	0.919	0	1.9	0
38	0	0	0	0.796	0	2.15	0
39	1	1	1	0.314	1	1.64	0
40	1	1	1	0.224	1	2.69	0
41	0	0	0	0.647	0	1.03	0
42	0	0	0	0.680	0	1.36	0
43	1	1	1	0.373	1	2.6	0
44	0	0	0	1.158	0	1.36	0
45	0	0	0	0.216	1	3.23	0
46	0	0	0	1.048	0	1.35	0
47	0	1	1	0.256	1	4.06	1
48	0	0	0	0.693	0	1.14	0
49	1	1	1	0.115	1	7.71	1
50	0	0	1	0.682	0	1.05	0
51	0	1	1	0.394	1	2.81	0
52	0	1	1	0.066	1	2.62	0
53	0	1	1	0.447	1	1.89	0
54	0	0	0	0.885	0	1.62	0
55	0	0	1	0.471	1	2.5	0
56	1	1	1	0.496	1	6.08	1
57	0	0	0	0.687	0	1.66	0
58	0	0	0	0.700	0	1.72	0
59	1	1	0	0.107	1	4.41	1
60	1	1	0	0.294	1	2.91	0
61	0	0	0	0.558	0	2.42	0
62	0	0	0	0.930	0	1.73	0
63	1	1	0	0.268	1	4.8	1
64	0	0	0	0.290	1	3.53	1
65	0	1	0	0.403	1	2.3	0
66	0	0	0	0.911	0	1.15	0
67	1	1	1	0.104	1	4.5	1
68	0	0	0	1.090	0	1.71	0
69	1	1	1	0.173	1	8.13	1
70	0	0	0	0.611	0	1.42	0
71	0	0	0	0.658	0	2.15	0
72	1	0	1	0.499	1	1.87	0
73	0	0	0	0.715	0	1.6	0
74	1	0	0	0.222	1	9.69	1
75	0	0	0	0.472	1	4.05	1
76	0	0	0	0.681	0	2.17	0

77	1	1	0	0.159	1	10.67	1
78	0	0	0	0.855	0	1.54	0
79	0	0	0	1.006	0	1.38	0
80	0	0	0	0.861	0	2	0
81	0	0	0	0.843	0	1.85	0
82	0	0	0	0.967	0	1.61	0
83	0	0	0	0.994	0	2.48	0
84	0	0	0	1.052	0	1.6	0
85	0	0	0	0.929	0	2.36	0
86	0	0	0	0.626	0	1.81	0
87	1	1	0	0.172	1	5.39	1
88	1	1	0	0.412	1	3.65	1
89	1	1	1	0.098	1	3.89	1
90	0	0	0	0.517	0	1.68	0
91	0	0	0	0.326	1	2.74	0
92	0	0	0	0.661	0	1.49	0
93	1	0	0	0.239	1	5.22	1
94	0	0	0	0.946	0	2.02	0
95	0	0	0	0.671	0	1.31	0
96	0	0	0	0.850	0	1.55	0
97	0	0	0	0.863	0	1.6	0
98	0	0	0	0.884	0	1.87	0
99	1	1	0	0.367	1	9.91	1
100	0	0	0	0.461	1	3.91	1
101	1	1	0	0.280	1	2.76	0
102	0	0	0	0.788	0	1.57	0
103	0	0	0	0.766	0	1.48	0
104	0	0	0	0.609	0	1.85	0
105	0	0	0	0.340	1	2.22	0
106	0	0	0	0.309	1	1.77	0
107	0	0	0	0.189	1	5.69	1
108	0	0	0	0.862	0	1.38	0
109	0	0	0	0.632	0	1.53	0
110	0	0	0	0.943	0	1.55	0
111	0	0	0	0.733	0	1.1	0
112	0	1	0	0.319	1	2.2	0
113	1	1	1	0.485	1	4.98	1
114	0	0	0	0.612	0	1.13	0
115	0	0	0	0.748	0	1.73	0
116	1	1	0	0.198	1	4.41	1

117	0	0	0	0.890	0	1.48	0
118	0	0	0	0.934	0	1.7	0
119	1	1	0	0.110	1	5.89	1
120	0	0	0	0.532	0	1.26	0
121	0	0	0	0.647	0	1.34	0
122	0	0	0	1.017	0	1.48	0
123	0	0	0	0.567	0	1.61	0
124	1	1	0	0.136	1	10.09	1
125	1	1	1	0.110	1	10.32	1
126	0	1	0	0.222	1	9..00	1
127	0	1	0	0.347	1	2.71	0
128	0	0	0	0.566	0	5.29	1
129	0	0	0	0.625	0	2.88	0
130	0	0	0	0.349	1	2.2	0
131	1	1	1	0.397	1	3.49	0
132	1	1	0	0.120	1	7.99	1
133	0	0	0	0.656	0	2.45	0
134	0	0	0	0.910	0	1.96	0
135	0	0	0	0.924	0	1.68	0
136	0	0	0	0.389	1	2.23	0
137	0	0	0	0.797	0	1.71	0
138	0	0	0	0.226	1	8.92	1
139	0	0	0	0.311	1	3.57	1
140	0	0	0	0.207	1	7.28	1
141	0	0	0	0.578	0	1.62	0
142	0	0	0	0.337	1	1.98	0
143	0	0	0	1.079	0	1.7	0
144	0	0	0	1.048	0	1.49	0
145	0	0	0	1.020	0	1.48	0
146	1	0	0	0.402	1	2.11	0
147	0	0	1	0.402	1	5.69	1
148	1	1	1	0.147	1	5.56	1
149	1	1	0	0.179	1	4.31	1
150	1	1	0	0.172	1	13.9	1
151	1	1	0	0.183	1	11.38	1
152	0	0	1	0.442	1	2.18	0
153	0	0	1	0.340	1	2.6	0
154	0	0	0	0.463	1	2.27	0
155	0	1	0	0.492	1	2.13	0
Maximum				1.232			

MRI: Magnetic resonance imaging; FDG: 2-[18F]-F-fluorodeoxyglucose; PET: Positron emission tomography; ECE: extracapsular extension; CN: central necrosis; Grouping: three or more contiguous confluent, SUVmean: mean standard uptake value. Extracapsular extension, central necrosis, and three or more contiguous confluent LRPNs are the characteristics of apparent positive nodes in MRI. All “1” in table represents positive while “0”, the negative.
